# A Rare Presentation of Severe Organophosphate Poisoning: A Case Report and Review of Literature

**DOI:** 10.7759/cureus.31497

**Published:** 2022-11-14

**Authors:** Anastasia E Ibrahim, Henrik Ghantarchyan, Thucminh Le, Ankur Bhagat, Bahareh Maknouni, Sarkis Arabian

**Affiliations:** 1 Intensive Care Unit, Arrowhead Regional Medical Center, Colton, USA; 2 Internal Medicine, Arrowhead Regional Medical Center, Colton, USA; 3 Critical Care, Arrowhead Regional Medical Center, Colton, USA

**Keywords:** suicide attempt, critical care, ddvp, poisoning, organophsophate

## Abstract

Organophosphates are chemicals commonly used as pesticides and work to inhibit acetylcholinesterase, leading to acetylcholine build up at muscarinic and nicotinic receptors throughout the body. Poisonings are often seen as small volume, chronic cases due to agricultural exposures, but can present as suicide attempts via organophosphate ingestion. Organophosphate poisonings, particularly when large volumes are ingested, require rapid and robust initiation of treatment. We present a case highlighting the appropriate management of profound organophosphate toxicity. We present a case of a 40-year-old female brought in by ambulance after purposefully ingesting two bottles of 100mL dichlorvos (DDVP), one of the largest volume organophosphate ingestions documented in the literature. She presented with severe salivation, diaphoresis and encephalopathy and was then intubated, requiring mechanical ventilation. She received multiple days of intensive care as she was treated with atropine, pralidoxime, intravenous fluids and vasopressors.

## Introduction

Organophosphates (OPs) are chemicals commonly used in pesticides. They are favored due to their broad spectrum effect against numerous types of agricultural pests and low cost. However, the use of these chemicals has also been banned and out of favor in the past two decades for their toxicity and teratogenicity to humans and animals. Systemic absorption of OPs can occur via inhalation or absorption through mucous membranes, skin, conjunctival and gastrointestinal exposure. For this reason, they have also been used as chemical weapons [1]. However, most exposures in the United States today occur in farm workers and their children over a chronic period of time due to occupational exposure [2]. Worldwide, there has been some success in limiting the use of organophosphates as pesticides, but accidental exposures and suicidal ingestions persist [1].

OPs are acetylcholinesterase inhibitors that bind acetylcholinesterase and prevent acetylcholine breakdown. They work at synaptic junctions within the autonomic nervous system, central nervous system and neuromuscular junctions. This leads to an accumulation of acetylcholine at these sites, saturating muscarinic and nicotinic receptors [2]. Over time, the bond between the organophosphate compound and acetylcholinesterase can become irreversible making administration of the antidote, 2-pralidoxime (2-PAM), paramount [2]. Activation of the muscarinic receptors leads to cholinergic effects including excessive secretions, diaphoresis, diarrhea, urination, and bronchoconstriction.

Activation of the nicotinic receptors can be lethal, particularly stimulation of the nicotinic receptors within the cerebellum which can lead to respiratory depression [1]. Acute exposures can also lead to chronic neuropsychiatric issues, such as motor impairment, psychosis, depression, memory and cognitive flexibility [1,3].

Current treatment for organophosphate poisoning is two-fold, which includes supportive care along with atropine and 2-PAM. 2-PAM is essential for survival, as it reactivates the previously organophosphate-bound acetylcholinesterase and allows for renewed reuptake of acetylcholine at the neuromuscular junction, reversing the life-threatening effects of the pesticide [4]. The best formulation of the drug and mechanism of administration are still being investigated, including increasing the ability to cross the blood-brain barrier by increasing lipophilicity and adjusting the polarity of N-oxime formulations. However, 2-PAM has been shown to free a majority of the organophosphate-bound acetylcholinesterase and restore activity of the enzyme [5].

## Case presentation

A 40-year-old female was transported by an ambulance to the Emergency Department (ED) after consuming two bottles of dichlorvos (DDVP) an hour prior to the transport. Patient presented with tachycardia, however no diarrhea or other hemodynamic abnormalities were noted on presentation. After detailed history taking, it was discovered that she had an extensive history of suicidal ideation with multiple suicide attempts, but no prior organophosphate use.

The patient was intubated immediately for worsening encephalopathy and risk of respiratory failure. An initial pH was 7.28, obtained on a venous blood gas. She received an initial dose of atropine 2 mg intravenous (IV) at the time of intubation to control severe secretions and was started on pralidoxime 500 mg/hr after consultation with a regional poison control center. Throughout her course in the ED, her acidemia worsened as her pH on an arterial blood gas (ABG) decreased to 7.21 and bicarbonate level was 14.5 mmol/L. 

**Figure 1:**
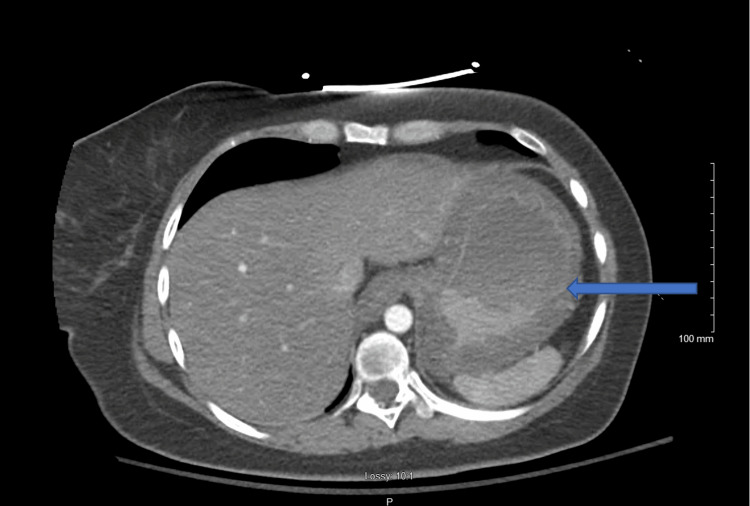
CT abdomen and pelvis with intravenous contrast with arrow signifying marked diffuse thickening of the stomach.

The second dose of atropine 2 mg IV was administered 1.5 hours after intubation to decrease excess oral secretion followed by intravenous administration of three liters of lactated Ringers in the ED. The patient progressively went into shock as her blood pressure decreased from 110/80 to 92/69 and was started on vasopressors. A computed tomography (CT) of the abdomen and pelvis at this time showed caustic esophageal injury and diffuse edematous thickening of the stomach (Figure 1). The Gastroenterology (GI) team was consulted for the concerning CT findings, and recommended against a nasogastric (NG) or orogastric (OG) tube, or a gastric lavage due to risk of perforation. She was then admitted to the Medical Intensive Care Unit (MICU).

Reconsultation of pharmacy revealed that our institution, a county hospital and level 1 trauma center, contained 12 grams of pralidoxime total, enough for 24 hours of treatment at a rate of 500 mg/hr. This is relevant given that although we are a large facility, we were still not prepared to handle such a large volume toxicity for an extended period of time. On the first day of her hospital course, all hospitals in the region were contacted and did not have stores of pralidoxime. However, on her second day of admission, the pharmacy was able to procure 24 grams total from two nearby academic centers and 12 grams from our hospital's main pharmaceutical distributor.

Upon admission to MICU, the patient was given another dose of atropine for continued oral secretions, bringing to a total of three atropine doses. Over the next two days, she became more hemodynamically unstable and acidotic with a pH of 7.05 with worsening shock. Vasopressor requirements increased to three agents at maximum doses. These included norepinephrine at 45 mcg/min, phenylephrine at 200 mcg/min, and vasopressin at 0.03 units/min. At this point she had received a total of 6.5 liters of fluids and was started on a sodium bicarbonate drip for severe acidosis. Poison control was again contacted, who noted that organophosphates can cause a delayed hypotension that can be poorly responsive to vasopressors. Additionally, there was no evidence in the medical literature supporting dialysis in cases of organophosphate poisoning. Nephrology also agreed with the lack of evidence of dialysis, therefore dialysis was not considered.

During her second day in the MICU, she had worsening anasarca. Her albumin level decreased to 1.5 g/dL. A total of 125 g albumin was administered during the day, with a vast improvement of both her edematous exam and her blood pressure, from 53/34 mmHg to 110/60 mmHg. 

Vancomycin and cefepime were also given for prophylaxis against possible fistula formation or translocation of bacteria due to caustic damage to her GI tract. After two days of treatment and negative blood cultures, these antibiotics were de-escalated to ceftriaxone and Flagyl for three more days for a total of 13 days of antibiotics. After three days into her hospitalization, the patient began having continued bouts of diarrhea with strong odors of organophosphates. Areas of skin exposed to diarrhea were erythematous but no ulcerative lesions were appreciated and a rectal tube was put in place to contain stool that was likely contaminated with DDVP. With the support and confirmation of the Organophosphate Exposure Hotline, we confirmed that isolation precautions were not required. She continued to hemodynamically improve during her ICU course from then on and was able to be weaned off vasopressors. Six days after her admission, she was successfully extubated and downgraded to the general medicine floor.

A barium swallow showed penetration without aspiration and CT abdomen and pelvis demonstrated moderate subcutaneous soft tissue stranding in the bilateral flank and posterior abdominal wall, however no evidence of perforation, free fluid, abscess or significant adenopathy. An upper endoscopy demonstrated mild linear erythema of the mid-esophagus with LA class B erosive esophagitis. However, the rest of the esophagus was otherwise normal. There was also severe, erosive and exudative gastritis noted throughout the stomach after biopsies of the antrum and body. 

After consistent evaluation over the next 72 hours with a one-to-one sitter and a 72-hour psychiatric hold due to danger to self, she continued to endorse no suicidal ideations, stated she intended to willingly stay in the hospital for treatment and expressed regret for ingesting organophosphates. The patient had an extensive hospital course with a significant recovery, with a successful discharge home to her family.

## Discussion

Respiratory failure is known as the most common cause of death in organophosphate poisoning, not only due to acetylcholine excess at the neuromuscular junction leading to weakened respiratory muscles, but also due to action at nicotinic receptors in the medulla, leading to decreased central respiratory drive [6]. However, severe metabolic acidosis has been found to be a poor prognostic factor in cases of organophosphate poisoning, especially in the setting of maintained respiration [6-8]. Many case studies over the years have shown the incidence of severe metabolic acidosis following organophosphate poisoning with an associated hemodynamic instability, oftentimes refractory to therapy [6,7,9]. In a report that examined four cases of severe organophosphate poisonings and measured hemodynamic changes via pulmonary artery catheter, the defining factor of each patient’s severe hypotension was a decreased systemic vascular resistance index, while cardiac output remained normal or increased. These parameters are most consistent with a distributive shock picture such as severe septic shock [6]. This severe distributive shock remained refractory to treatment, and persisted even after norepinephrine was administered in this study. The severely reduced peripheral vascular resistance can be accounted for through the action of acetylcholine at muscarinic receptors on peripheral blood vessels, leading to vasodilation and a distributive shock effect. Additionally, there may be a compounded vasodilatory effect of parasympathetic fibers in the autonomic nervous system, which responds to activation of muscarinic receptors as well [6]. Finally, previous studies have shown that there is also a significantly higher level of nitric oxide seen in patients with organophosphate poisoning than those without, leading to further vasodilation [10]. These mechanisms may contribute together to the severe hypotension seen in this case and those documented prior. Its refractory nature may be due to prolonged acetylcholine action at these muscarinic receptors due to irreversible binding of acetylcholinesterase to the ingested organophosphate.

Lactic acidosis should be considered as a possible etiology of severe metabolic acidosis in this case, due to severe hypoperfusion in the setting of refractory hypotension. However, it is still unclear if organophosphates themselves have an effect on increasing lactate [11]. There is also mention of a rare case of renal tubular acidosis in the setting of organophosphate poisoning that completely resolved with atropinization, however this is much less likely to be the cause of the acidosis in this case as renal complications of organophosphate poisoning are almost unheard of [9]. In this case, the patient’s severe metabolic acidosis was initially refractory to treatment with lactated Ringer's fluid resuscitation and sodium bicarbonate treatment. In fact, cases in the literature describe improvement of metabolic acidosis only after treatment with pralidoxime and atropine, suggesting the organophosphates themselves provide a primary mechanism for this severe metabolic acidosis. However, treatment with fluid resuscitation and sodium bicarbonate may still be supportive until the action of the organophosphates ingested are reversed [6,8].

Studies have also cited ketoacidosis as a possible cause of refractory metabolic acidosis in the setting of organophosphate poisoning. Cases reviewed presented with hyperglycemia and ketonuria without a history of diabetes mellitus, with some reporting glucose levels after poisoning up to the 800s [8]. Animal studies have shown reversible hyperglycemia after acute administration of organophosphates that usually peaks two hours after administration and lasts up to six days. It is thought that the etiology of this hyperglycemia is the stimulation of glycogenolysis and gluconeogenesis by organophosphates directly via activation of glycogen phosphorylase and phosphoenolpyruvate carboxykinase, essential enzymes in the reaction. Additionally, oxidative stress and activated inflammatory pathways by the ingested organophosphate are thought to promote insulin resistance, contributing to a patient’s hyperglycemia [12]. It is less likely that the hyperglycemia is due to pancreatic damage and thus an absence of insulin due to the loss of beta islet cells, as autopsy done after organophosphate poisoning showed intact pancreas despite extreme hyperglycemia [8]. 

Finally, hypokalemia, which was as low as 2.3 mmol/L in this case, has also been routinely documented in other case studies of severe organophosphate poisoning, and suggests an effect of organophosphates in potassium distribution between the intracellular and extracellular space [8]. However, the alkalotic effect of this hypokalemia is outweighed by the associated ketoacidosis and possible additional lactic acidosis in these patients.

In this case, however, we also suspect there may be a hypovolemic component to the patient’s shock, particularly after the severe secretions she presented with. Although her hypotension was refractory to aggressive fluid resuscitation, the patient was found to be increasingly edematous, while remaining intravascularly depleted, demonstrating clinical signs of third spacing of fluid into the interstitial space. In light of her decreased albumin levels and third-spacing, albumin repletion was administered, with the aim to increase oncotic pressures intravascularly and encourage fluid in the interstitium to return to the intravascular system and aid in correcting her hypotension. In fact, only after continuous treatment with pralidoxime and administration of albumin did her hypotension become responsive to treatment, her mean arterial pressure (MAP) began to improve and her vasopressor requirement began to decrease.

As similarly seen in cases of OP poisoning, the patient in this case presented with refractory metabolic acidosis, elevated lactate, hypokalemia and hyperglycemia, though to a milder level than those reported in the literature. Her hyperglycemia with a blood glucose range of 220-290 mg/dL and trace ketones in her urine point to a ketoacidotic source of metabolic derangement, while her mildly elevated lactate of 1.97 mmol/L on her admission day points to a lactic acidosis contributing to her metabolic acidosis. All these contributing factors began to trend towards normal as she received aggressive fluid resuscitation, sodium bicarbonate and pralidoxime treatment. Gastrointestinal losses can also be considered as a possible contributing factor. However, it is also important to note that her metabolic acidosis continued to worsen in the absence of diarrhea and only after she began having diarrhea did her clinical status begin to improve, most likely due to her ability to expel the ingested organophosphate. 

Dialysis was also considered as a possible treatment modality for clearing organophosphate after her ingestion. However, there are opposing opinions on the efficacy of hemodialysis throughout the literature. Some cited efficacy but only after 163 hemoperfusions, in one such case. However, discussions of the efficacy of plasmapheresis as a treatment option are on the rise and warrant further investigation [13-15]. In light of these findings, her severe hypotension and a poison control consultation which recommended against dialysis, the patient did not receive hemodialysis in conjunction with pralidoxime and atropine treatment. 

While providing supportive care and addressing severe metabolic acidosis in the setting of OP poisoning, the curative arm of treatment is administration of pralidoxime and atropine. Research suggests that organophosphates inhibit acetylcholinesterase on red blood cells and at synapses throughout the body, leading to acetylcholine build-up at muscarinic and nicotinic receptors [4,13]. This binding reaction of the organophosphate molecule to acetylcholinesterase becomes irreversible over time, in a process known as “aging” [4]. Depending on the type of organophosphate ingested, the time to aging differs. Acetylcholinesterase inhibited by diethyl organophosphorus has a longer time to age, with an approximate half-life of 31 hours, whereas their dimethyl organophosphorus counterparts have a shorter time to age, with an approximate half-life of 3.7 hours [5]. However, the regeneration reaction to increase acetylcholinesterase availability can take longer. For this reason, treatment with the reversal agent oximes, namely pralidoxime, is more effective in the cases of poisonings with diethyl formulations than dimethyl [4,16-18]. Pralidoxime not only works to reverse the acetylcholinesterase-organophosphate reaction, if not yet aged, but also decreases the availability of inactive acetylcholinesterase to bind to unbound organophosphate molecules, important to prevent progression of symptoms and the deleterious effects of the poisoning that could ultimately lead to death. Literature review has also shown that in the case of ingestion of dimethyl organophosphates, as seen in the case of this patient, pralidoxime is often only effective for about 12 hours of treatment due to rapid aging, making early administration vital to success. 

Atropine is another critical arm of the treatment of organophosphate poisoning. As an anticholinergic drug, atropine reverses the associated cholinergic symptoms in OP poisoning, such as severe secretions, by competitively binding the muscarinic receptors and decreasing availability of these receptors for free acetylcholine to bind [17]. This can be lifesaving in decreasing symptoms such as excessive secretions and bronchospasm. Additionally, at higher doses, atropine has been shown to have an effect in prevention against aging of the organophosphate reaction by 28-60% at 1 mM. However, clinical doses of atropine are much lower and used to quell secretions rather than prevent aging of the reaction [19]. In addition, continuous and high doses of atropine can lead to decreased intestinal motility due to its antimuscarinic effects, increasing the time to expelling the organophosphates from the GI system and thus overall systemic exposure to the pesticides [6]. Thus, atropine should be given in the setting of severe secretions and should be concomitant with pralidoxime treatment in order to both address the active symptoms as well as to prevent the irreversible aging of the reaction. Additionally, atropine should be given prior to pralidoxime initiation to avoid worsening of muscarinic-mediated symptoms of pralidoxime alone [20]. 

As mentioned above, the patient had consumed two bottles of 100 mL each, totaling 200 mL of 53.7% DDVP. According to the Centers for Disease Control and Prevention (CDC), oral ingestion of greater than five ounces of 5% of DDVP can result in coma, inability to breathe, and death [21]. Our patient had ingested an amount significantly more than the lethal dose. Finally, due to this large ingestion, there was concern for high gastrointestinal perforation risk due to the caustic nature of the pesticides. There are differing thoughts in the literature about how to address gastric damage due to organophosphate ingestion, although most agree that induction of vomiting or diarrhea to eliminate the substance should be avoided. Activated charcoal can be administered but has not been shown to demonstrate significant benefits and gastric lavage has been used in certain cases [6,20]. The patient did have an elevated white blood cell count with bandemia initially, and was started on broad-spectrum antibiotics due to risk of bacterial translocation in the case of a perforation or possible fistulas formed from the caustic ingestion. Nasogastric tubes, orogastric tubes and gastric lavage were avoided in this case due to the risks of perforation in the case being greater than the benefits they would provide. However, CT abdomen and later esophagogastroduodenoscopy (EGD) confirmed no perforation, though demonstrated severe caustic injury to the esophagus and stomach. Thus her elevated white count, while still plausibly due to translocated bacteria, could also be accounted for by severe inflammation due to tissue damage incurred by the caustic ingestion. Her antibiotics were then de-escalated and she was started on a liquid diet by the end of her admission.

Limitations 

Our study is limited by this singular case, providing some difficulty in generalizing an exact protocol due to low power, as well as site-specific availability of medications and time to treatment. The successful treatment of this patient’s OP poisoning hinges on the time to initiation of treatment and availability of pralidoxime, whereas treatment of her metabolic acidosis and respiratory failure were supportive measures. It is difficult to account for changes in timing of initial dose of pralidoxime and how a later initial dose may have altered the course of treatment. Finally, this case is also limited by treatment style and clinical judgment to decide when to adjust course of treatment and when to stop pralidoxime treatment. In order to combat errors or bias in decisions based on clinical judgment, our team heavily relied on literature review of similar cases and also collaborated in a multidisciplinary fashion with poison control, pharmacists and physicians from other institutions and our own to guide our clinical decision making.

## Conclusions

This case demonstrates the importance of pralidoxime and atropine as mainstays of OP poisoning treatment and the key to reversing severe metabolic acidosis and shock. Shock can be multifactorial in these cases, so aggressive fluid resuscitation and sodium bicarbonate administration are important adjunct treatments, though supportive in nature and not curative. Albumin can also help address third spacing in the setting of hypovolemia. Additionally, time to initial pralidoxime dose is crucial to the success of treatment so we recommend having at least twelve grams of pralidoxime in hospital pharmacy stores at all times in order to initiate treatment quickly. Finally, it is important to have an anesthesia or intubation team aware of the patient in case of airway compromise and to have a low threshold for intubation, as not only can secretions and bronchospasm affect the airway but central respiratory effects are the most common cause of death in these patients.
